# Polylactide-Based Materials: Synthesis and Biomedical Applications

**DOI:** 10.3390/molecules28031386

**Published:** 2023-02-01

**Authors:** Marek Brzeziński, Malgorzata Basko

**Affiliations:** Centre of Molecular and Macromolecular Studies, Polish Academy of Sciences, Sienkiewicza 112, 90-363 Lodz, Poland

Polylactide (PLA) is a biocompatible polyester that can be obtained by polycondensation of lactic acid or the ring-opening polymerization (ROP) of lactide. It is worth noting that its unique properties are also related to the existence of two enantiomeric forms: poly(l-lactide) (PLLA) and poly(d-lactide) (PDLA), which form a supramolecular complex. This complex is called a stereocomplex, and its formation leads to enhanced thermal and mechanical properties, decreased degradation time or drug release. All these advantageous features predispose PLLA to applications in the biomedical field. Various materials are prepared from PLA matrix, such as nanoparticles (NPs), microparticles (MPs), nonwovens, implants, hydrogels, 3D-printed materials, etc. However, there is an unmet need for novel and more effective materials that can deliver their cargo to the desired site of the body. The main aim is to overcome drug resistance to antibiotics or anti-cancer drugs by appropriately designing PLA-based materials. In this regard, supramolecular materials are required since their unique structure allows them to self-heal or release the drug with slight environmental fluctuations (pH, temperature, light, etc.). It is also important to evaluate the in vivo stability of biosafety of these novel materials before their administration to humans. Due to the above-mentioned issues, there are goals that still need to be achieved, despite the tremendous applications of PLA-based materials in biomedicine; moreover, artificial intelligence may be useful in designing these materials in the near future. 

This Special Issue presents the original research and reviews articles describing the synthesis and application of PLA-based materials. In this regard, Razuvaeva et al. [1] reported the preparation of NPs composed of methoxy poly(ethylene glycol)-*b*-poly(d,l-lactide) copolymers loaded with the anticancer drug: oxaliplatin. The core–corona structure was obtained after the nanoprecipitation of copolymers. It was shown that the higher amount of hydrophobic block results in a decrease in the core–corona interface and a lowering of the drug loading. The size of drug-loaded NPs ranges from 19 to 27 nm, and its highest loading is for NPs built from copolymers with shorter hydrophobic blocks, which is related to the low hydrophobicity of the drug. In addition, hybrid NPs composed of PLGA and a lipid block for the delivery of paclitaxel against lung cancer cells was proposed by Lirdprapamongkol et al. [2]. The self-assembled NPs were uniform, with a size of around 100 nm and a negative charge on their surface. Those NPs were tested against A549 attached and floating cells. Both cell lines show dose-dependent cytotoxicity; treatment of the floating cells (anoikis resistant) by NPs increased efficiency, since the 71.6-fold decrease in IC_50_ compared to the free drug was observed. Several mechanisms may be responsible for paclitaxel resistance of A459 cancer cells; however, the enhanced efficiency of nanocarriers is probably related to their ability to deliver paclitaxel into the cell interior. Ayari-Riabi [3] proposes a nanoparticle for the preparing unit vaccine that can be used against the vector-borne disease leishmaniasis. This tropical disease, and its most common form, Cutaneous leishmaniasis, causes skin infections. To overcome this problem, the poly(d,l-lactide)-based NPs were obtained by nanoprecipitation and mixed with *L. major* histone H2B (*L. major* H2B) protein, and they acquired immunogenicity. The adsorption of proteins on the surface of NPs (adsorption capacity: 2.8 (±0.24% *w/w*) induces the shift in charge from negative to positive, while the size of NPs increases from 270 to 340 nm. Such particles release H2B protein in a sustained way, and this feature facilitates the prolonged activity of the encapsulated protein. To test their efficiency, BALB/c mice were infected with *L. major* parasite, and the following formulations were administered: H2B protein/CpG-ODN7909 adjuvant, H2B protein/PLA-NPs, and H2B protein alone. The humoral and cellular immune response is induced for a combination of H2B protein with CpG-ODN7909 or PLA-NPs, respectively. Most importantly, the footpads of mice were unoccupied with necrosis, with fewer parasites on their surface in the group treated with H2B protein/PLA-NPs compared to the control. This proves that such a PLA-based system can be an anti-leishmanial vaccine.

Due to their biocompatibility, PLA-based materials can also be used as implants or dressings. In this regard, Wulf et al. [4] propose to cover cochlear implants with PLLA coating (loaded with diclofenac-DCF) along with medical-grade silicone as a matrix (loaded with dexamethasone-DMS) to achieve sustained release of these anti-inflammatory drugs. This is especially important after implantation since the long-term release of those drugs may cause undesired tissue reactions. To prepare the desired material, the silicone surface was treated with O_2_ plasma in the presence of (3-glycidyloxypropyl)trimethoxysilane. Subsequently, the surface was spray-coated with PLLA with amino functionalities. This material structure leads to totally different release profiles of encapsulated drugs. The PLLA layer on the silicon surface decreases the release of DMS; however, it accelerates the release of DCF. This dual-release profile will enhance the patient’s comfort after implantation. Furthermore, the impedance measurements were performed, and since PLA is an insulator, the masking on the surface of the research electrode contacts should be applied during the coating process. Those promising materials are planned to be investigated in vivo. On the contrary, the in vivo investigation of PLA biocompatibility and biodegradation after subcutaneous implantation on the lateral surface of the neck of a horse was described by Ferraz et al. [5]. The implants were constructed from commercially available PLA (Ingeo 3251D, NatureWorks) by hot pressing with the size of 1 cm^2^ and 1 mm thickness. The histochemical examination was performed 24, 28, 34, 38, and 57 weeks after implantation. The plasma fibrinogen level was constant, indicating a negligible inflammatory process. The foreign body response was detected until 38 weeks. Most importantly, SEM analysis indicated the occurrence of the biodegradation process since the implant’s surface became rough and the pore diameter increased. Interestingly, the polymer fragmentation after 57 weeks was significant; therefore, the implant cannot be removed and analysed, indicating PLA degradation. This process is complicated, since several factors must be considered during the degradation process, such as d-monomer content, crystallinity, molar mass, and especially conditions of this process. However, the proposed approach paved the way for the use of commercial grade PLA in veterinary because the chosen PLA was not a medical grade one. 

Electrospinning (ES) [6] and the air-jet spinning technique (AJS) [7] are the most relevant techniques for the preparation of PLA fibres. Pinese et al. [6] combined the ES process with chemical crosslinking to prepare nanofibers resistant to degradation. Star-shaped PDLLA was synthesised and equipped with triethoxysilylpropyl (TPES) end groups to achieve this aim. These end groups are subsequently used during the ES process to crosslink the resulting nanofibers with 360 to 780 nm diameters. It relies on the reaction of hydrolysis of TPES and the formation of silanol groups that can condense to form a network. Several parameters, such as polymer dilution, molecular weight, and the addition of low-molecular-weight PTES-functionalized PLA to prepare the material able to withstand 6 months of degradation. Most importantly, the obtained materials exhibit acceptable biocompatibility with murine and mouse fibroblasts cells lines that predispose their application in biomedicine. Alvarez-Perez et al. [7] use the AJS technique to develop nanocomposite fibre scaffolds for tissue bone regeneration. The first step towards this aim was the synthesis of ZrO_2_ NPs by the hydrothermal method. Those NPs (0.1 or 0.5 g) were added to commercially available PLA (NatureWorks), and the AJS technique was suitable for obtaining composite fibres with an average diameter of 395 nm. The human foetal osteoblast cell attachment on the surface of obtained mats was at the suitable level, whereas the highest viability was for the nanocomposite with 0.5 g of nanoceramics; therefore, those fibres were used for the biomineralisation essay. It was shown that PLA/ZrO_2_ exhibit a higher concentration of calcium precipitates on their surface than those composed solely of PLA. These good pro-osteogenic properties indicate their potential for bone regeneration application. 

PLA also has several drawbacks, such as brittleness or low impact strength; therefore, the methods of its properties modification are desirable. In this respect, the blending of PLLA with polycaprolactone (PCL) and poly(3-hydroxybutyrate-co-3-hydroxyvalerate) (PHBV) is proposed to prepare materials for orthopaedical applications by Dalgarno et al. [8]. The main goal was to modify the creep behaviour of PLLA to fulfil the requirements for materials applied in bone regeneration. Twin screw extrusion is suitable for combining this material, and the analysis by differential scanning calorimetry (DSC) proved their effective blending. The ternary blends PLLA/PCL/PHBV (80/10/10) were ideal since the increased modulus, strength, and elongation at break can be achieved. Therefore, the biomaterials with the desired properties can be obtained by adjusting the ratio of macromolecules in the resulting composite. Alternatively, starch/PLA (NatureWorks) blends with oil polyols, polyethylene glycol (PEG), and citric acid (CA) as modifiers were proposed by Zhao et al. [9]. Reactive blending leads to the preparation of desired materials with the elongation at break, and impact strength is greatly improved compared to pure PLA. This affected the esterification reaction between CA, PEG, and starch. Interestingly, the value of tan δ in the low-frequency region shows gel-like behaviour indicating a crosslinking reaction. Moreover, the resulting material was hydrophilic in contrast to hydrophobic PLA. A different approach was shown by Bojda et al. [10], which investigated the influence of topology on shear to induce the crystallisation of PLA. Therefore, the high-molecular-weight 4-arm and 6-arm PLLAs were synthesised, and their properties were compared with linear counterparts. As expected, the influence of the macromolecular structure and molecular weight on the shear-induced crystallisation was determined. Another way to modify the properties of PLA-based materials is the addition of nanofillers such as carbon nanotubes (CNTs), as proposed by Makowski et al. [11]. To achieve this aim, the ES method was used to prepare nonwovens with 0.1 wt.% CNTs and 5 and 10 wt.% oligomeric linear ladder poly(silsesquioxane)s (LPSQ) as additives. The porous fibres with a diameter from 0.68 to 3.5 μm were obtained, and their size decreased with the addition of CNTs and LPSQ. Most importantly, mechanical parameters can be greatly improved due to the presence of additives. It was shown that a combination of 10 wt.% LPSQ-COOMe and 0.1 wt.% of MWCNT lead to a 2.4-times increase in the tensile strength and elongation at maximum stress. In contrast, the flame retardancy of PLA (4032D, NatureWorks) can also be modified by appropriate additives, as shown by Zhang et al. [12]. Firstly, 9,10-dihydro-9-oxa-10-phosphaphenanthrene-10-oxide (DOPO) was reacted with endic anhydride (EA), and the resulting product was melt blended with commercially available PLA. Using vertical combustion (UL-94), the limiting oxygen index (LOI) tests indicate that the addition of ammonium polyphosphate (APP) to the composite is required to achieve an increased LOI value of 32.2% and a UL V-0 rating. Their good dispersion causes the synergistic effect of these two additives in the PLA matrix and the formation of an intumescent protective layer on the surface of the resulting material, which results in high thermal stability and flame retardancy. 

The reviews in this Special Issue cover many topics, focusing mainly on strategies for extending the range of polylactide-based materials applications in biomedicine. The properties of PLA homopolymers, their copolymers, blends, and composite materials prepared for the manufacture of devices suitable for bone fixation were reviewed by Naseem et al. [13]. In conclusion, the authors indicated that current and prospective research should focus mainly on the search for polyester materials with appropriate mechanical properties, adjustable degradation time, modifiable brittleness, modified degradation chemistry to avoid excess acid degradation products, and improved interactions between the device and native tissue. Furthermore, the contribution concerning the chemical modification of the PLA backbone by introducing reactive structures that enabled the graft copolymers synthesis in the next step was presented by Coudane et al. [14]. After presenting the methods enabling the functionalization of the PLA backbone, the results of grafting various polymer segments onto the polyester backbone were discussed. By reacting the anhydride or epoxide groups incorporated into the PLA chain, various polymers, including cellulose derivatives, polyesters, polyamides, natural rubbers and PMMA, were combined with PLA. The main applications of these PLA graft copolymers in the field of environmental protection and biomedicine are proposed. In addition, the preparation of drug carriers, hydrogels and fibres in which supramolecular interactions occur in the cyclodextrin/polymer system has been reviewed by Kost and Brzeziński et al. [15]. Cyclodextrins are a group of cyclic oligosaccharides with a specific structure that enables the formation of inclusion complexes with various molecules through non-covalent host–guest interactions. Due to these specific association abilities, cyclodextrins are used in the pharmaceutical industry to increase the solubility and stability of drugs. By combining biocompatible polymers with cyclodextrins, various multifunctional materials have been developed. The presented work is divided into four sections which describe the drug delivery systems based on poly(lactide), poly(ɛ-caprolactone), poly(ethyleneglycol), and poly(sacharides). The paper discusses the release of different biologically active substances, including antibiotics, vitamins, hormones, enzymes, anticancer drugs, non-steroidal anti-inflammatory drugs and physiologically active lipid compounds. In contrast, Kim [16] reviews strategies for applying stereocomplex PLA in drug delivery systems and biomedicine. Synthesis and processing methods that enabled the production of therapeutic carriers in various forms (stereocomplex micelles, self-assembly nanoparticles, emulsions, hydrogels, and 3D-printed materials) were discussed in the article. Presented fields for potential stereocomplex PLA material applications concerned drug delivery systems, anti-cancer therapy, tissue engineering, and anti-microbial activity. Moreover, systems based on crosslinked polyesters (polymeric networks) are widely used in the area of drug delivery, wound healing, tissue engineering or medical implants. Methods of PLA crosslinking by applying different types of irradiation, i.e., high-energy electron beam or gamma irradiation and UV light, were reported by Bednarek et al. [17]. Finally, the progress of research on PLA nanocomposites containing synthetic organic nucleators (arylamides, hydrazides and 1,3:2,4-dibenzylidene-d-sorbitol) and biological nucleators (orotic acid, humic acids, fulvic acids, nanocellulose, cyclodextrins) in the context of their biomedical application was discussed by Kowalewska et al. [18]. The main role of these additives was to improve the rate of PLA of crystallization kinetics (nucleation and crystal growth) through supramolecular interactions based on hydrogen bonds or host–guest effects. Supramolecular interactions operating in those blends play a very important role in the properties of those novel hybrid materials.

In summary, the Special Issue entitled “Polylactide-Based Materials: Synthesis and Biomedical Applications” compiles the most recent research works on the modification of PLA materials by supramolecular interactions or the addition of nanofillers. It highlights the biomedical applications of PLA-based materials in drug delivery, tissue regeneration, bone fixation, etc. It is anticipated that acquired knowledge will lead to better understanding of the structure–properties relationship and will help to develop novel advanced strategies in biomedical and pharmaceutical field.
